# Correlation Between Endoscopic and Radiological Data in Rhino-Orbital-Cerebral Mucormycosis (ROCM)

**DOI:** 10.7759/cureus.101461

**Published:** 2026-01-13

**Authors:** Arjun A Graison, Ashfaque Ansari, Ajinkya Raverkar

**Affiliations:** 1 Otolaryngology - Head and Neck Surgery, Mid and South Essex NHS Trust, Southend-on-Sea, GBR; 2 Otolaryngology - Head and Neck Surgery, Mahatma Gandhi Mission (MGM) Medical College and Hospital, Aurangabad, IND; 3 Otolaryngology - Head and Neck Surgery, Index Medical College, Hospital and Research Center, Indore, IND

**Keywords:** covid-19, covid-associated mucormycosis, diagnostic nasal endoscopy, head and neck radiology, rhino-orbito-cerebral mucormycosis (rocm)

## Abstract

Background

The invasive fungal illness known as rhino-orbito-cerebral mucormycosis (ROCM) is associated with significant morbidity and fatality rates. The increasing burden of the disease and lack of uniformity in guidelines related to its management have led to an increasing case fatality rate. The main objective of the study was to compare and corroborate the cross-sectional imaging findings of CT and MRI scans with those of intra-operative endoscopic findings.

Methods

This retrospective study was conducted at a tertiary care centre of Mahatma Gandhi Memorial Hospital in Aurangabad, India. Each patient was treated with systemic antifungal therapy and surgical debridement following cross-sectional imaging. The findings on radiology and intraoperative endoscopies were compared, and data were analysed.

Results

Radiological assessment demonstrated high sensitivity, specificity, and positive predictive value (generally >90%) across most anatomical sites when compared with intra-operative endoscopic findings. The maxillary sinus was the most commonly involved sinus in almost 97% of cases, followed by the ethmoid sinuses. The middle turbinate was the most commonly involved among the three turbinates. Diagnostic performance was lower for the superior turbinate, with a positive predictive value (PPV) of 79.3%. Chi-square analysis showed no statistically significant difference between radiological and endoscopic detection across the anatomical sites assessed.

Discussion

Cross-sectional imaging plays a vital role in the preoperative assessment and management of mucormycosis. MRI scan gives better delineation of orbital involvement and soft tissue extension, as mucormycosis is well known for angioinvasion. However, despite overall concordance between imaging and endoscopic findings, reduced sensitivity in specific anatomical subregions, including the superior turbinate, intraconal compartment, and select maxillofacial soft-tissue spaces, indicates that early disease may be under-represented on imaging alone. These findings support the role of targeted endoscopic exploration to detect occult disease during surgical management. Imaging is also a useful tool in the follow-up of the disease after the patient's discharge on step-down therapy.

Conclusion

Cross-sectional imaging with CT and MRI provides a valuable adjunct for preoperative mapping in ROCM and works synergistically to delineate disease extent, including orbital and deep soft-tissue involvement that may not be clinically apparent. As imaging guides surgical planning and identifies anatomically high-risk regions that would otherwise not be explored, CT and MRI should be performed in all cases of ROCM. However, early disease involvement in several anatomically concealed areas may be missed on cross-sectional imaging; therefore, endoscopic correlation of hidden regions remains essential to ensure complete disease clearance.

## Introduction

Mucormycosis is a notable opportunistic infection caused by a fungus of the order *Mucorales *[1]. This condition is characterized by rapid progression from the nasal mucosa and sinuses, influenced by factors such as vascular invasion, congenital bony anomalies, bone erosion, and spread via the nasolacrimal ducts, lymphatics, and neurovascular bundles into adjacent structures, including the orbit and cerebral tissue [2].

Individuals with compromised immune systems, e.g., those who have diabetes mellitus, solid organ transplants, hematological malignancies, and those who are undergoing prolonged immunosuppressive therapy, have a significantly higher risk of contracting this infection [3,4].

Throughout the COVID-19 pandemic, there was a significant rise in instances of COVID-19-associated rhino-orbital-cerebral mucormycosis (ROCM). The increase can be attributed to several factors, including the widespread use of corticosteroids, contaminated ventilator masks, and infected hospital oxygen systems, along with industrial oxygen sources that have surfaced as new causes for this condition [5]. Compared to global-scale statistics, the estimated prevalence of mucormycosis in India is seventy times higher [6].

The approach to invasive fungal sinusitis generally requires the swift commencement of antifungal treatment alongside careful surgical measures. Timely identification is essential for successful treatment results [7]. Diagnostic techniques like potassium hydroxide (KOH) mounts are utilized to verify fungal presence, whereas modalities of imaging, such as CT and MRI, offer crucial insights into the presence, extent, involvement, and intensity of the infection [8].

Comprehensive imaging of mucormycosis is essential for assessing the involvement of craniofacial tissues, the skull base, orbital, vascular structures, and cerebral compartments. Radiological data are crucial for guiding surgical decision-making by assessing disease location, extent, severity, and potential risks and problems associated with surgical intervention.

Despite its central role, CT and MRI may underestimate early extrasinus or soft-tissue spread, particularly in hidden regions that are not easily accessible on routine nasal endoscopy. In contrast, intra-operative endoscopic exploration of hidden areas can reveal subtle mucosal or submucosal involvement that is not apparent radiologically. Understanding the degree of agreement between these modalities is therefore clinically important, as it helps determine which regions can be reliably assessed on imaging and which require routine endoscopic exploration during surgical debridement.

The aim of this study was to evaluate how well cross-sectional imaging (CT and MRI) correlates with intra-operative endoscopic findings across predefined sinonasal, orbital, and maxillofacial anatomical sites, and to identify regions where imaging is less reliable and routine endoscopic exploration is warranted.

This study, involving 98 patients diagnosed with invasive fungal sinusitis at Mahatma Gandhi Mission Medical College and Hospital, seeks to investigate the relationship between endoscopic and radiological results to improve comprehension and management of this critical illness.

## Materials and methods

Study design

This retrospective observational study was conducted at Mahatma Gandhi Mission Hospital, Aurangabad, in the department of Ear, Nose, and Throat (ENT) surgery from April 2021 to October 2022. Patients admitted to the department during the study period were screened for eligibility, and 98 patients were recruited for the study.

Inclusion Criteria and Exclusion Criteria

Patients included in the study were those diagnosed with post-COVID-19 mucormycosis confirmed either by KOH mount samples or histopathological analysis, and who subsequently underwent definitive endoscopic surgical excision.

Patients were excluded from the study if they had a confirmed diagnosis of post-COVID-19 mucormycosis by biopsy or KOH mount but did not undergo definitive surgical excision due to secondary underlying comorbidities. Additionally, cases involving mixed fungal infections identified on KOH mounts or histopathology were also excluded. Patients who lacked cross-sectional imaging in the local imaging system before definitive surgical intervention were excluded. Finally, patients who did not provide consent for endoscopic surgical excision were not included in the study.

Methodology and Consent

All KOH mount proven cases or histopathologically proven cases of post-COVID-19 mucormycosis admitted for definitive surgery were subjected to detailed history taking, including local examination with nasal endoscopy and systemic examination.

All recruited patients were informed of the prognosis of the disease and treatment modalities, including surgery and systemic antifungal therapy, followed by oral step-down therapy. The consequences and complications of invasive fungal ROCM were explained to them.

In order to find radiology-endoscopy correlation, for each predefined sinonasal, orbital, and maxillofacial anatomical site, CT/MRI reports and intra-operative endoscopic findings were coded in a binary fashion as "involved" or "not involved".

Imaging protocol 

CT Protocol

CT examinations were performed on a 64-slice multidetector scanner (Fujifilm Corporation, Tokyo, Japan). Axial images were acquired with 5-mm slice thickness and 5-mm intervals using the B50 algorithm, with additional 1-mm high-resolution reconstructions (B60) for evaluating fine bony detail. Non-contrast CT was used in all patients, as its primary role in ROCM is the assessment of bony erosion and sinus involvement; contrast-enhanced CT was reserved only for specific clinical indications.

MRI Protocol 

MRI was performed on a 1.5-Tesla scanner (Philips Multiva, Philips Healthcare, Best, The Netherlands) using a 16-channel neurovascular coil. Multiplanar imaging was obtained with axial, coronal, and sagittal T1W, T2W, and T2-fat-suppressed sequences, along with diffusion-weighted imaging (DWI) and susceptibility-weighted imaging (SWI) as required. Post-contrast T1-fat-suppressed sequences were acquired when renal function allowed. MRI served as the primary modality for assessing extrasinus soft-tissue extension, orbital involvement, and perineural or cavernous sinus spread.

All scans were reviewed by a consultant radiologist for formal reporting.

History and Clinical Examination

All patients presented with suspicion of mucormycosis underwent detailed clinical evaluation and history to rule out underlying comorbidities like diabetes mellitus, systemic immunosuppression, recent use of chemotherapeutic agents, and use of immunosuppression drugs. The examination included nasal endoscopy, focusing on the three nasal endoscopy passes (first, second, and third). A clinical note was made of the initial spread of the disease, and subsequently, the cross-sectional imaging was planned before surgical intervention.

**Figure 1:**
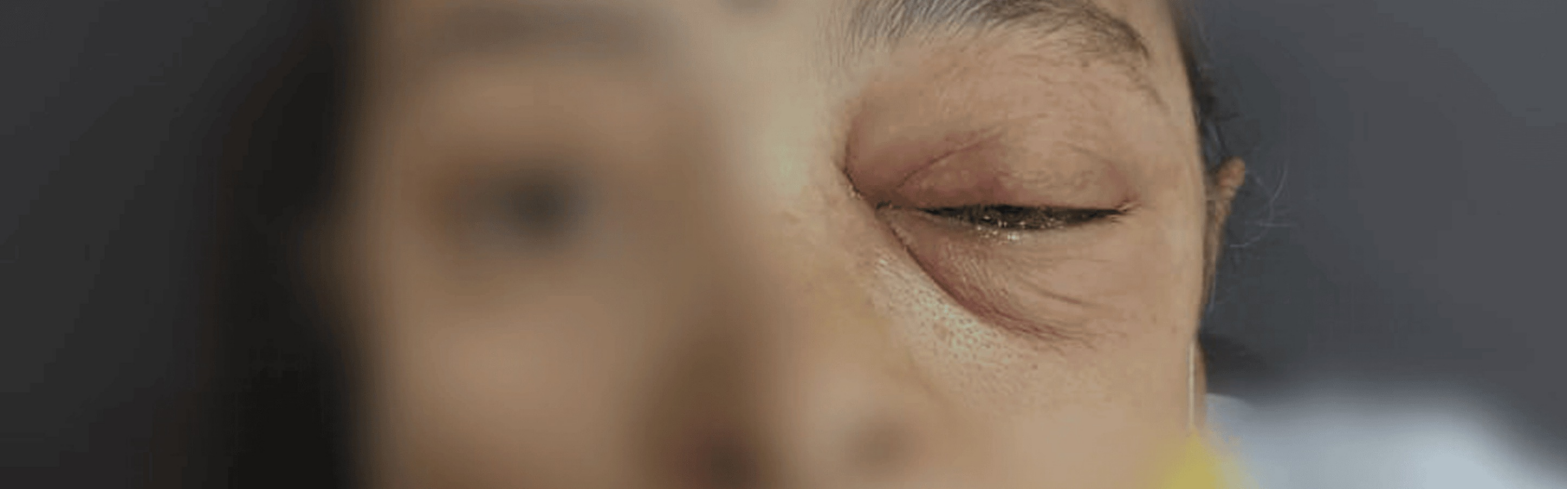
Clinical examination showing left eye exophthalmos with ptosis suggestive of orbital involvement with mucormycosis

**Figure 2:**
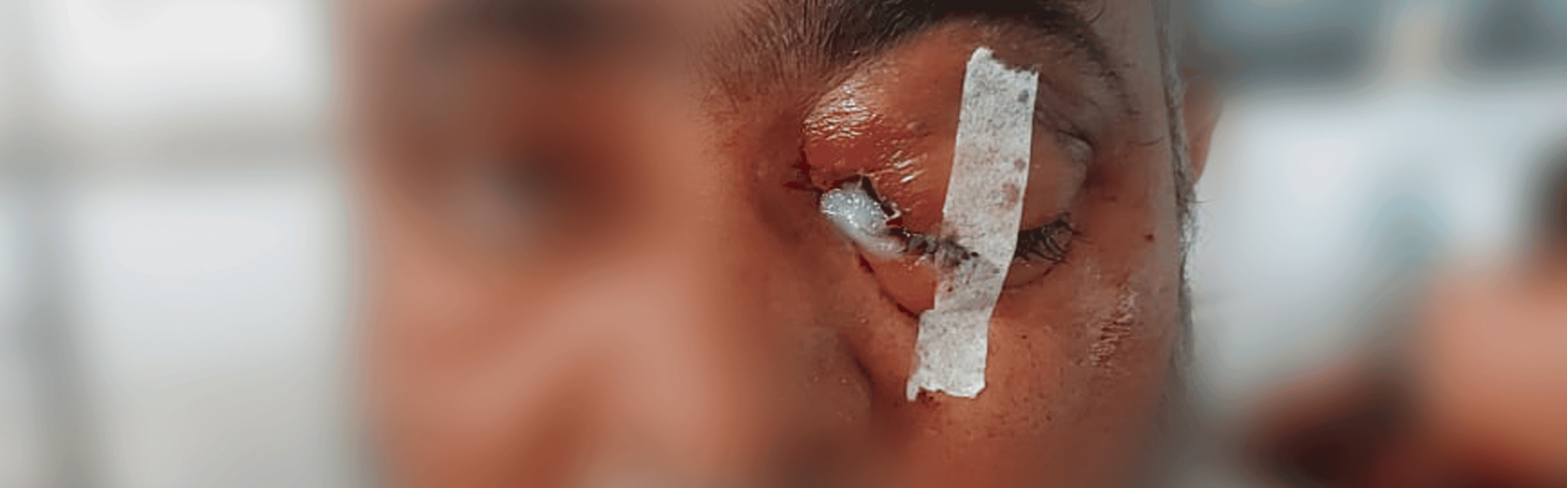
Clinical image of left eye ecchymosis and ptosis showing extensive orbital involvement due to invasive mucormycosis

**Figure 3:**
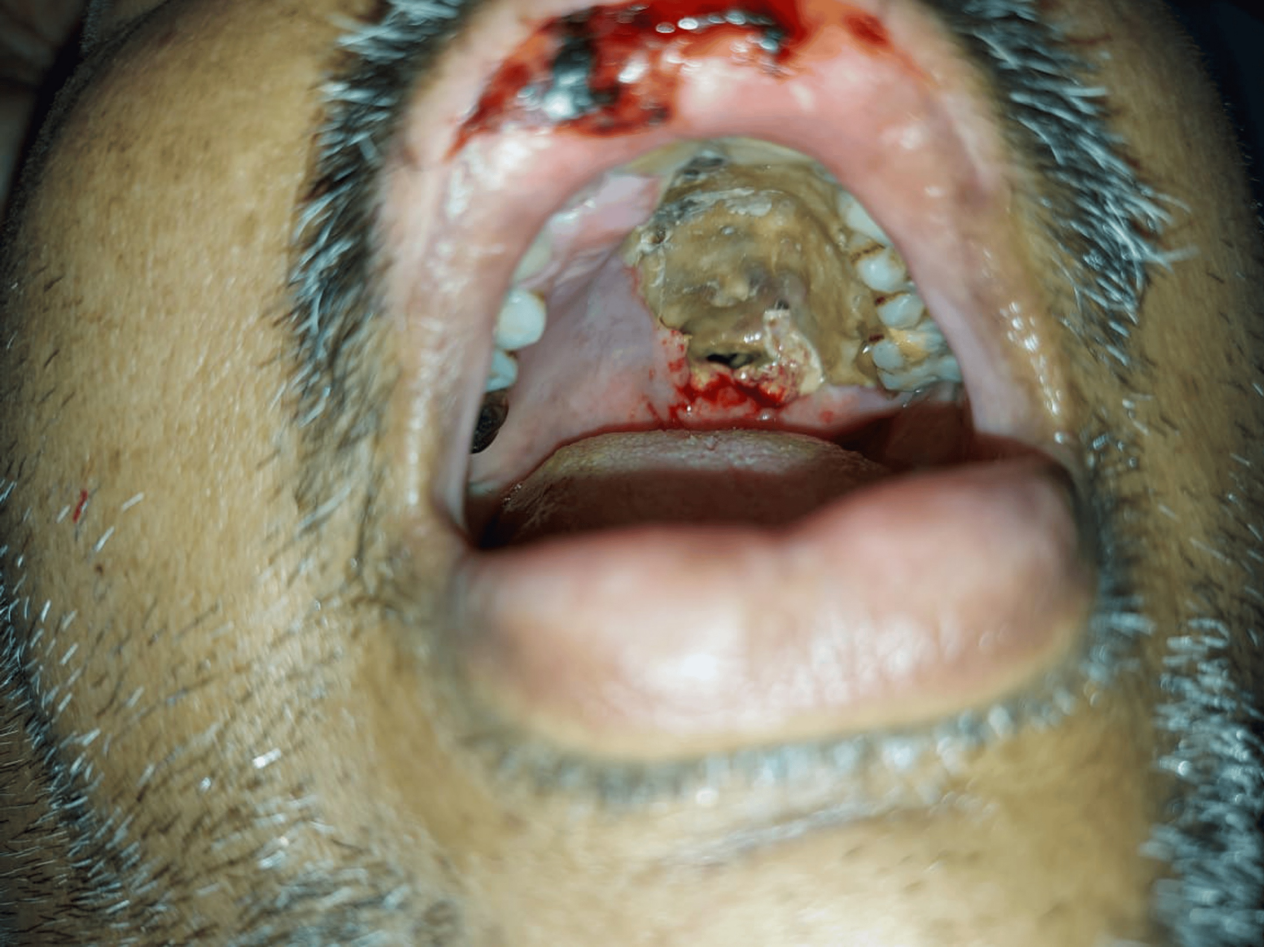
Clinical examination of oral cavity showing left palatine bone involvement and erosion by invasive mucormycosis

**Figure 4:**
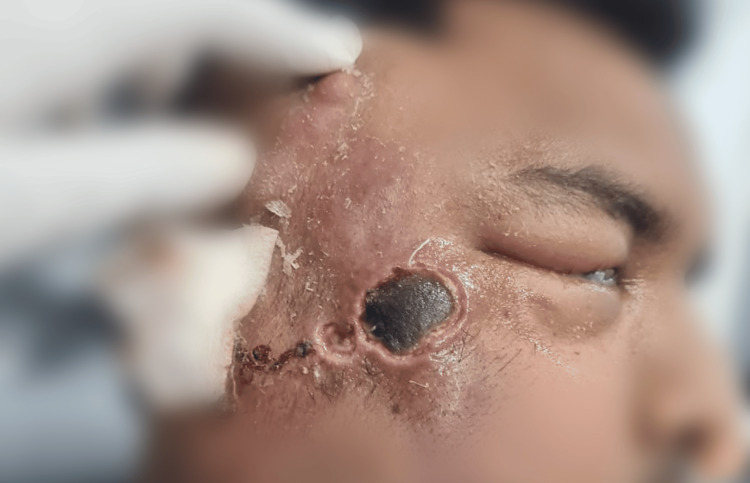
Clinical image showing right zygoma and overlying skin involvement with right eye ptosis and ecchymosis with invasive mucormycosis

Other relevant clinical examinations included assessment of visual acuity, colour vision, relative afferent pupillary defect (RAPD), and cranial nerve (CN)** **V dermatome assessment for parasthesia (Figure 1-4).

Investigations

All routine hematological and biochemical investigations required as a baseline to start antifungal treatment and surgical intervention were carried out on initial admission. Blood glucose and kidney function tests were closely monitored during the hospitalisation stay.

Cross-sectional imaging in the form of contrast-enhanced computed tomography scan (CECT) and contrast-enhanced magnetic resonance imaging (CEMRI), such as gadolinium-enhanced magnetic resonance imaging scan of the nose and paranasal sinuses with the brain, was performed to evaluate the preoperative disease load. After the definitive surgical intervention, endoscopic evaluation of the postoperative cavity was performed on alternate days to determine the healing status of the cavity and to pick up early residual disease, if any.

**Figure 5:**
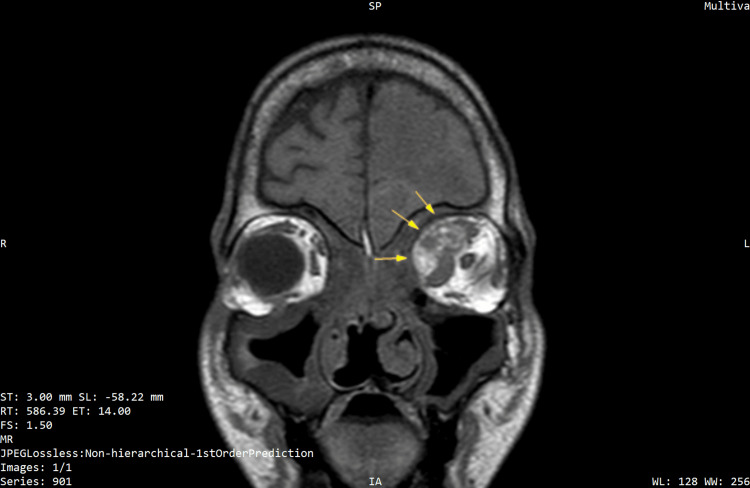
T1W coronal image showing the involvement of mucormycosis in the left orbit with lamina papyracea erosion (demarcated by yellow arrows)

Postoperative cross-sectional imaging in the form of CEMRI was done on the third day to look at residual status, if any, and to monitor the intracranial disease extension, if any (Figure 5).

Treatment

Systemic Treatment

All suspected cases of invasive ROCM were started on systemic amphotericin B. Depending on the recent kidney function test and availability, patients were started on liposomal amphotericin B or conventional amphotericin B according to the patient's weight. In patients allergic to amphotericin B or having severe kidney function test derailment, systemic antifungals from the azole group, like oral posaconazole, were started as a systemic therapy.

Systemic antifungal therapy with early surgical intervention plays a pivotal role in the management of ROCM. Amphotericin B is the drug of choice for invasive mucormycosis. It attaches to ergosterol, the primary constituent of the fungal cell membrane. It has hydrophilic and hydrophobic regions. It results in acidification of the fungal interior with cytoplasmic precipitation and cell death.

Posaconazole is used as a step-down therapy or is also indicated in patients with a reaction to amphotericin B. Posaconazole is a well-tolerated extended-spectrum triazole exhibiting in vitro and in vivo activity against *Mucorales*. It is available in intravenous as well as oral gastro-resistant tablets. The oral formulation is used in patients as step-down therapy.

Adequate glycaemic control prevented fungal spore production even after surgical debridement. Regular blood sugar monitoring and working with the endocrine department were done to manage it. Patients were given either oral hypoglycaemic agents or systemic insulin to take control of their blood sugar.

Surgical Intervention

Active liaising with the anesthetic team was of prime importance in times of emergency ENT crisis observed during the rampant spread of the ROCM in the COVID-19 pandemic. Patients were taken to the operating theatre as early as possible after adequate stabilisation of hematological parameters.

**Figure 6:**
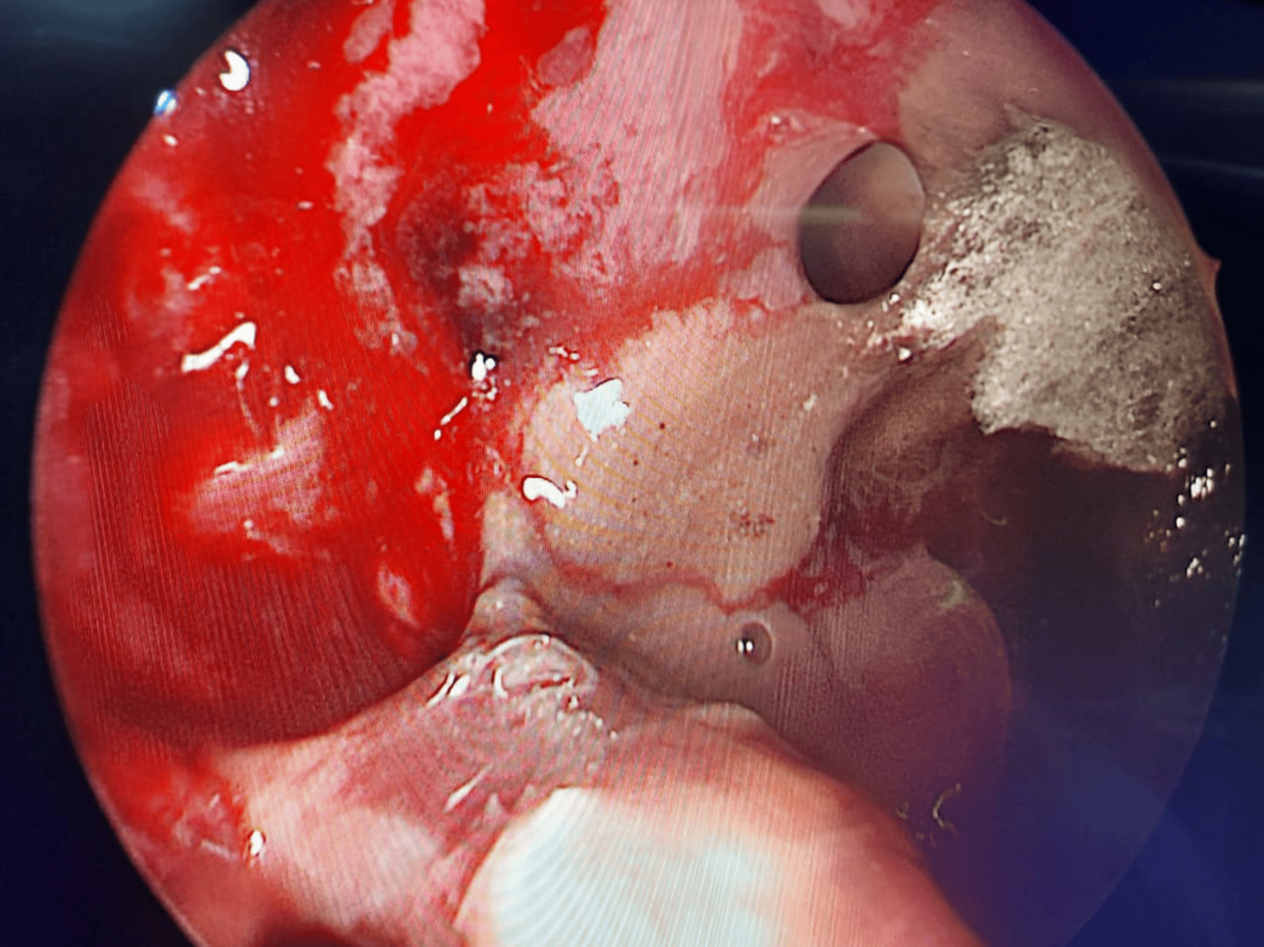
Fungal elements of mucormycosis seen in the pterygopalatine fossa on the left side on endoscopy

Three anatomical regions were identified through clinical experience as critical reservoirs for hidden fungal infection. These included the anterior inferior region of the maxillary sinus, the pterygopalatine and infratemporal fossae (Figure 6), and the lamina papyracea and orbital regions, especially when clinical signs indicated eye involvement.

Access to the anterior inferior portion of the maxillary sinus was achieved using one or a combination of approaches: the Caldwell-Luc method, the modified Denker's procedure, or an entirely endoscopic surgical technique.

Discharge and Step-Down Therapy

After clinical and radiological evidence of disease clearance, patients were discharged with follow-up appointments initially every week for a month and later, according to the individual patient profile. Post-discharge radiological scan in the form of CEMRI was done after three months for an initial six months, followed by six-monthly scans to monitor the disease process closely.

Posaconazole step-down therapy was given on discharge with a 300 mg/day dose to be continued for the initial six months. Close monitoring of liver function tests was done to monitor the adverse effects of the step-down therapy.

Statistical analysis

For each anatomical site, sensitivity, specificity, positive predictive value (PPV), and negative predictive value (NPV) of radiological assessment were calculated using intra-operative endoscopic findings as the reference standard. Agreement between radiological and endoscopic detection (involved vs not involved) was assessed using chi-square tests, with p<0.05 considered statistically significant. Statistical analysis was performed using SPSS (IBM Corp., Armonk, US).

## Results

In our sample of 98 cases, most participants were males, accounting for 80.4% (n=79)** **of the total cases, while females made up only 19.4% (n=19). Most participants were in the age group of 51 to 60 years, making up 36.7% (n=36) of our total cases.

Paranasal sinuses involvement

The paranasal sinuses were the anatomical sites that were most frequently affected bilaterally among all the 22 sites observed by both radiological and endoscopic observations. The ethmoid sinus was most commonly seen to be affected bilaterally (in 77.6% cases, n=76) in radiological examination, followed by the sphenoid sinus (in 75.5% cases, n=74). Radiologically, unilateral involvement of these two spaces was seen in 15.3% (n=15) and 14.3% (n=14) of cases, respectively, with 10.2% (n=10) and 7.1% (n=7) of cases showing no sphenoid and ethmoid sinus involvement, respectively. However, on endoscopic examination, all cases showed ethmoid sinus involvement, while the sphenoid sinus was spared in only 4.1% (n=4) of the cases.

**Figure 7:**
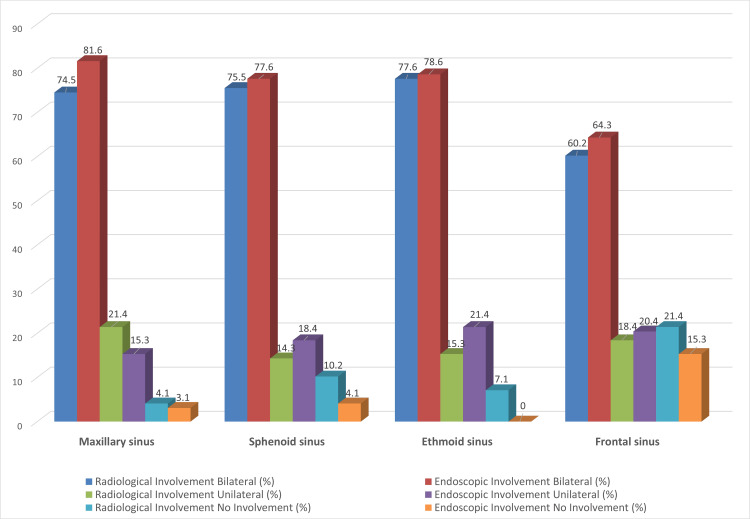
Comparison of the radiological and endoscopic findings in the following areas of paranasal sinuses

Radiological investigations revealed mucormycosis bilaterally present in only 74.5% (n=73) of cases in the maxillary sinus, whereas on endoscopy, the proportion increased to 81.6% (n=80). However, no involvement was seen in 4.1% (n=4)** **and 3.1% (n=3) of cases by radiological and endoscopic findings, respectively (Figure 7).

**Figure 8:**
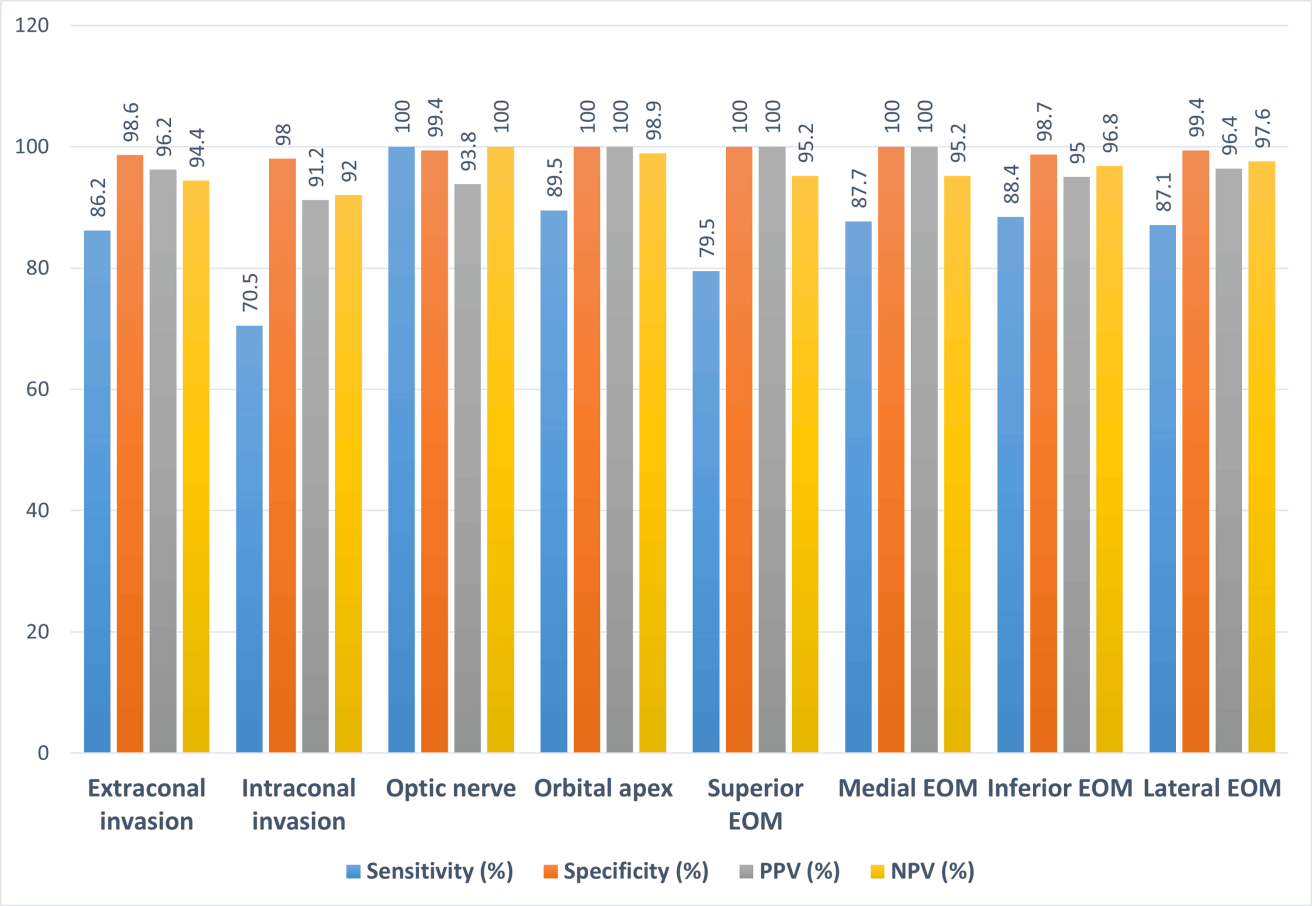
Diagnostic accuracy of radiological investigations with respect to endoscopic findings for intra-orbital regions EOM - extraocular muscle

On computing the diagnostic accuracy statistics of radiological testing to detect the presence of mucormycosis disease in the paranasal sinuses, we find that the test has very high sensitivity and specificity. The test's sensitivity was more than 90% in all four spaces and bilaterally, so the test showed 100% specificity for all spaces except for the left frontal sinus. A near-perfect positive predictive value implies that the presence of mucormycosis on radiological findings almost guarantees the presence of the disease in the space indicated and warrants exploration to remove the disease. However, negative predictive value was less than satisfactory in all four paranasal sinuses and was seen to be less than 70% for the maxillary and ethmoid sinuses - this suggests that the absence of disease on radiological scans does not reliably exclude disease in these regions (Figure 8).

Nasal turbinate involvement

Compared to the paranasal sinuses, the involvement of the three turbinates was less frequent. The inferior turbinate was the most frequently involved, with the presence of the disease seen in 77.6% (n=76) of cases on endoscopic exploration, of which 46.9% (n=46) had bilateral disease, and 25.5% (n=25) had only unilateral disease.

The superior turbinate was least frequently involved with the presence of disease seen in only 32.7% (n=32) of cases on endoscopy, of which 29.6% (n=29) had bilateral disease, and 3.1% (n=3) had unilateral involvement. Interestingly, however, radiological investigations suggested the presence of mucormycosis on the unilateral superior turbinate in 14.3% (n=14) of cases.

**Figure 9:**
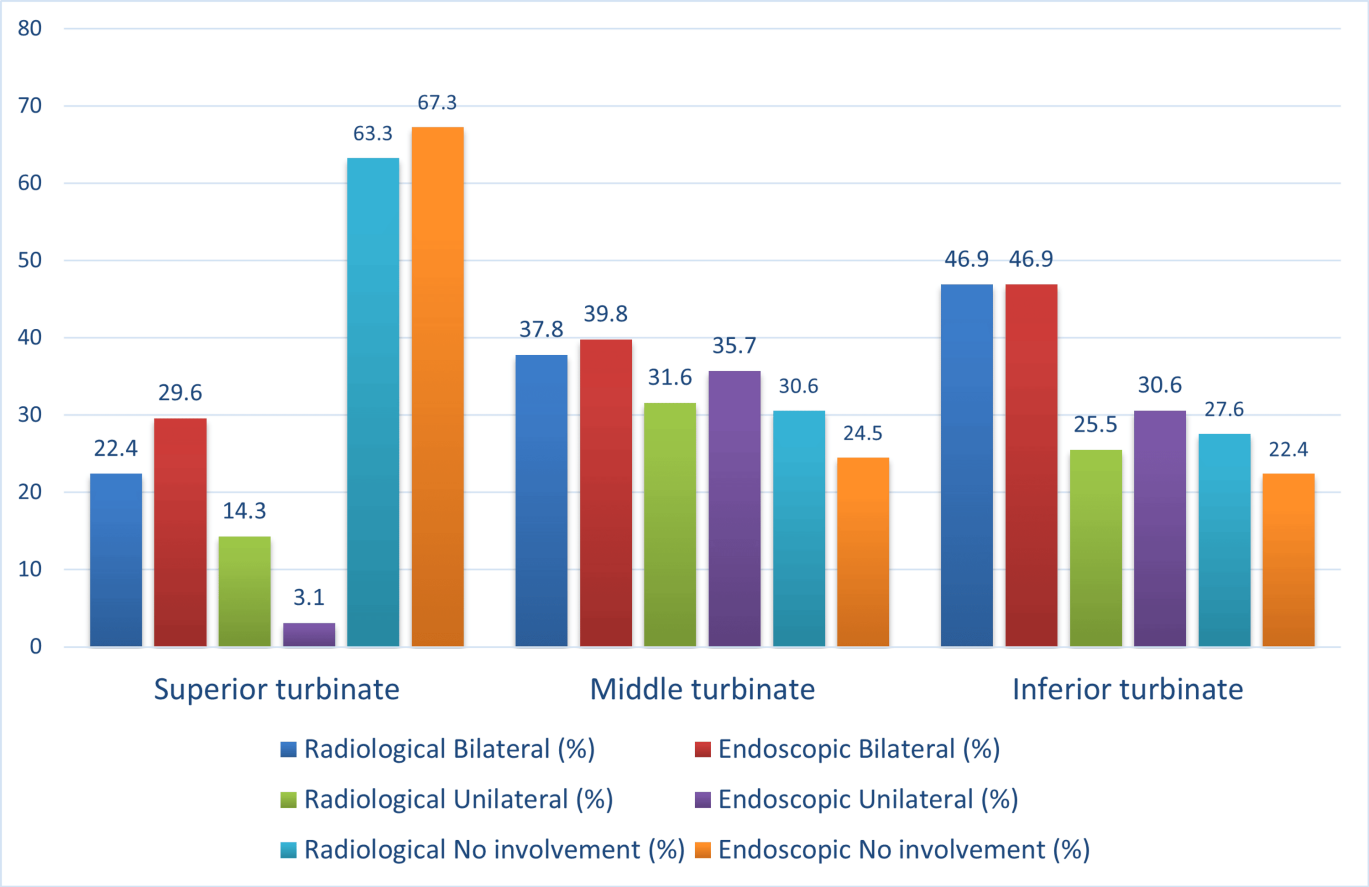
Comparison of the radiological and endoscopic occurrences of ROCM in the turbinates ROCM - rhino-orbito-cerebral mucormycosis

The middle turbinate was seen to be affected in 75.5% (n=74) of cases, of which 39.8% (n=39) had bilateral disease, and 35.7% (n=35) had unilateral involvement. Radiological investigations also revealed a similar proportion of involvement in the middle turbinate (Figure 9).

False positives were not seen in the middle turbinates, whereas they were seen in both the superior and inferior turbinates. Thus, the positive predictive value of radiological investigations for the middle turbinates was 100%. However, this was not the case for the other two spaces. For inferior turbinates, the PPV was 97.4%. The analysis for the inferior turbinate also revealed high sensitivity and specificity of more than 90% (93.4% and 95.9%, respectively).

**Figure 10:**
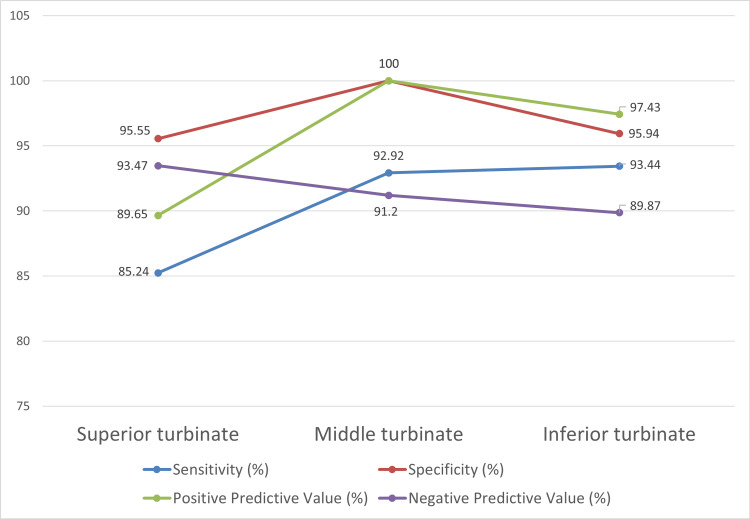
Diagnostic accuracy metrics for nasal turbinates

The PPV for superior turbinates was low at 89.65%. The sensitivity of this space was also low at 85.2%, which implies certain disease areas were missed on radiology (Figure 10).

Intra-orbital area involvement

Intra-orbital involvement was seen in a relatively lower proportion of cases when compared to nasal turbinates and paranasal sinuses. Extraconal involvement was seen most frequently in 42.8% (n=42) of cases, of whom 16.3% (n=16) had bilateral and 26.5% (n=26) had unilateral disease, which was followed by involvement of the medial extraocular muscle as seen in 39.8%(n=39) of cases, of whom 18.4%(n=18) had bilateral and 21.4% (n=21) had unilateral disease.

Endoscopically, the optic nerve and orbital apex were the least observed areas affected with mucormycosis in the intra-orbital region in our sample of cases, with the disease seen in only 14.3% (n=14) of cases for each site. Only one person had bilateral optic nerve involvement, and five cases had bilateral orbital apex involvement. Among the extraocular muscles, the lateral extraocular muscle (EOM) was least commonly affected in our sample, with disease present in 22.4% (n=22) of cases, of whom only nine cases (i.e., 9.2% proportion) had bilateral involvement.

False positives were not seen in the orbital apex, superior and medial extraocular muscles, and false negatives were not seen in the optic nerve. All the other regions showed false positive or negative findings.

**Figure 11:**
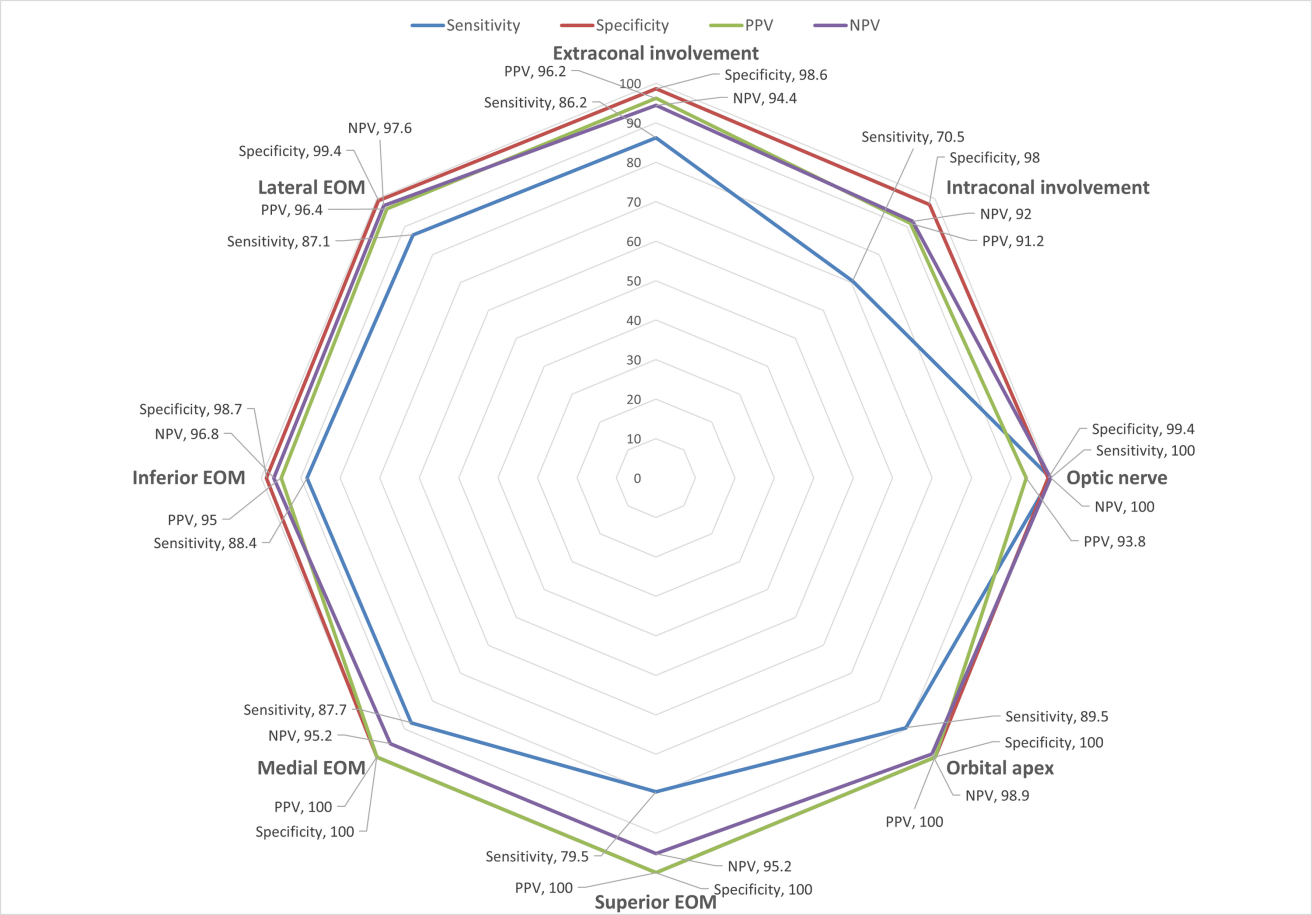
Diagnostic accuracy metrics for intra-orbital regions PPV - positive predictive value; NPV - negative predictive value; EOM - extraocular muscle

The diagnostic accuracy of radiological investigations with respect to positive and negative findings on endoscopy is good for the intraocular areas except for the superior extra-ocular muscles and intraconal space, which have poor sensitivity at 66.7% (for superior EOM), 78.3% and 61.9% on the right and left side respectively for intraconal involvement due to a relatively large number of false negatives in our sample (Figure 11).

Other areas in the maxillofacial region

On endoscopic findings, the other anatomical sites other than those mentioned earlier were involved in 20.4% (n=20) to 31.6%(n=31) of cases, much lower than paranasal sinuses and nasal turbinates, and comparable to the involvement observed in the intra-orbital areas. The masticatory space was seen to be most frequently involved in 31.6% (n=31) of the participants, with almost half of them, 13.3% (n=13) of all participants having bilateral involvement. A similar proportion of bilateral involvement was observed in the retro-antral pad of fat (18.4%, n=18) and pre-maxillary fat (12.2%, n=12), whereas the other areas, such as pterygopalatine fossa and fissure, the subcutaneous space, and the pre-maxillary fissure, showed the presence of bilateral disease to a much lower degree.

These findings are corroborated overall by the findings from radiological investigations, which show a slightly lower proportion of involvement across most areas. Only in the case of pre-maxillary fat, CT or MRI indicated disease presence in 27.4% (n=27) of cases, which was slightly higher than the findings of endoscopy at 20.4% (n=20). Further analysis of the diagnostic accuracy of radiological findings would reveal more details about the same.

Many false positive cases (n=15) were seen in the pre-maxillary fat region. This brings down the positive predictive value of a positive radiological finding in the pre-maxillary fat region to as low as 28.6%, implying that a positive radiological finding does not indicate finding the disease on endoscopic exploration.

**Figure 12:**
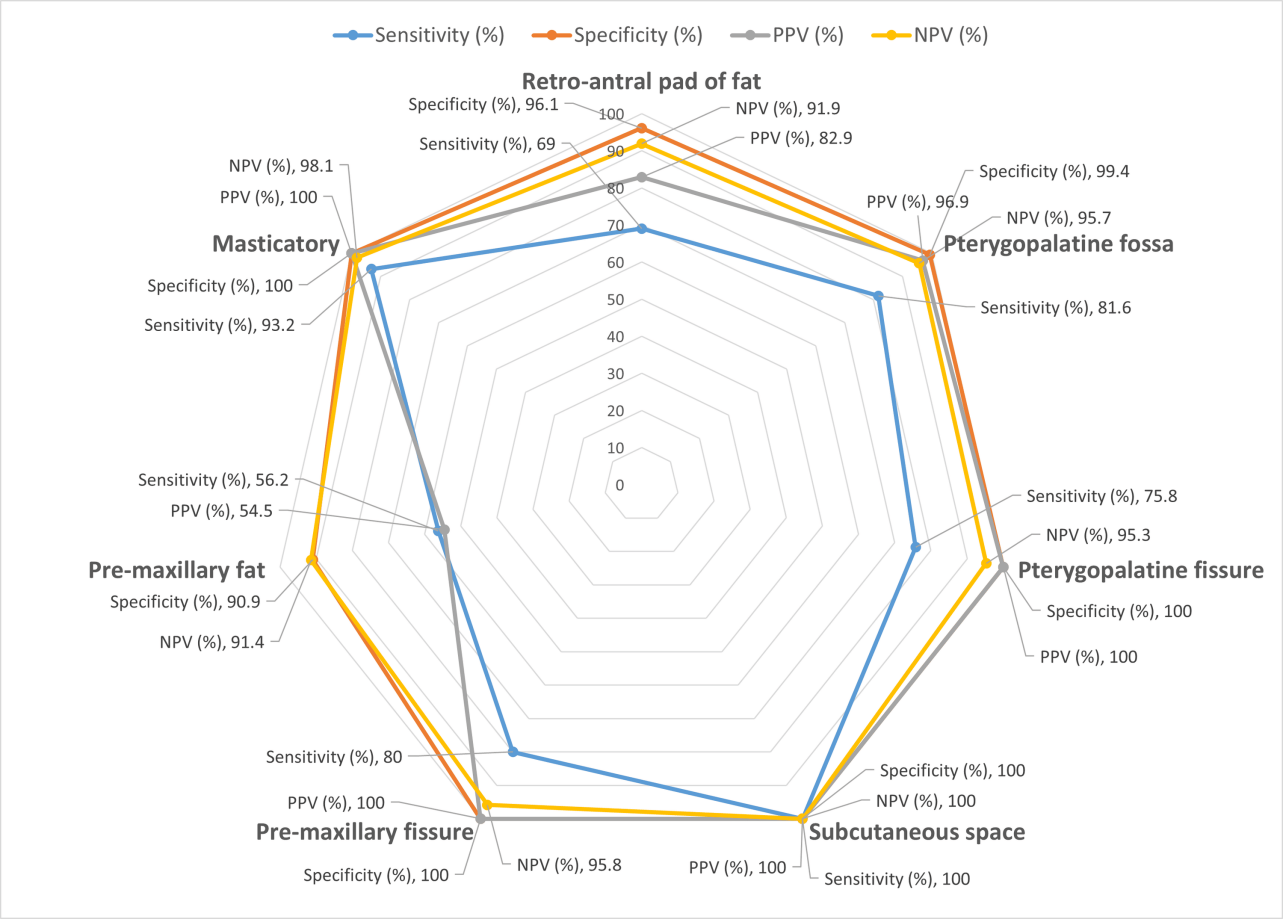
Diagnostic accuracy metrics for maxillofacial anatomical sites (compared to endoscopic findings) NPV - negative predictive value; PPV - positive predictive value

The diagnostic accuracy of radiological investigations (Figure 12) against a gold standard of endoscopic findings was relatively low in these anatomical sites, except for the masticatory space, which showed a high sensitivity of over 90% on either side and a specificity of 100%, and subcutaneous space, which showed a complete corroboration as mentioned earlier. Overall, the sensitivity of CT and MRI in these regions was quite low (except for the two spaces mentioned earlier), implying that a negative radiologic scan cannot sufficiently rule out the presence of disease, and endoscopic exploration of the site is warranted during surgery.

Comparison of radiological and endoscopic involvement across anatomical sites

To statistically corroborate these anatomical correlations, a chi-square analysis was performed comparing radiological versus endoscopic detection across all anatomical sites. The results are summarised in Table 1.

**Table 1:** Comparison of radiological and endoscopic involvement across anatomical sites using chi-square test EOM - extraocular muscle

Affected areas (includes left and right sides of each area)	No of sinuses found affected on radiology	No of sinuses found affected on endoscopy	Chi-square	p-value
Maxillary sinus	85	88	0.1713	0.679
Ethmoidal sinus	83	87	0.3529	0.5525
Sphenoid sinus	85	89	0.3979	0.5282
Frontal sinus	69	74	0.3926	0.5309
Superior turbinate	29	30	0.000	1.000
Middle turbinate	53	57	0.1818	0.6698
Inferior turbinate	59	62	0.0837	0.7724
Extraconal involvement	26	29	0.1003	0.7515
Intraconal involvement	17	22	0.5096	0.4753
Optic nerve	8	7	0.000	1.000
Orbit apex	8	9	0.000	1.000
Superior EOM	15	20	0.5541	0.4566
Medial EOM	25	29	0.2283	0.6328
Inferior EOM	20	22	0.0301	0.8622
Lateral EOM	14	15	0.000	1.000
Retroantral fat compartment	17	21	0.2924	0.5887
Pterygopalatine fossa	16	19	0.1385	0.7097
Subcutaneous space	13	13	0.000	1.000
Pterygopalatine fissure	12	16	0.3738	0.541
Premaxillary fissure	14	17	0.1527	0.696
Premaxillary fat pad	16	16	0.000	1.000
Masticatory space	21	22	0.000	1.000

Statistical comparison using chi-square testing showed no significant radiology-endoscopy discordance in any of the anatomical sites examined (p>0.05 throughout). This supports the study's primary objective by confirming that CT/MRI broadly correlates with operative findings and can be used as a dependable preoperative mapping tool. However, the lack of statistical significance should not be interpreted as perfect equivalence. Several regions demonstrated practical, clinically relevant mismatches, especially the superior turbinate, intraconal space, superior EOM, and pre-maxillary fat, highlighting that imaging may underrepresent early soft-tissue spread in these areas. These findings validate the need for routine endoscopic exploration of such anatomical sites even when imaging appears unremarkable.

## Discussion

ROCM was an epidemic that led to significant morbidities and mortality, especially in Southeast Asia and the Indian subcontinent. The treatment protocols embarked on to tackle this fatal condition were different, in different parts of the same country, due to limited resources and scarcity of systemic antifungals at the time of the epidemic. The most common reason employed for the rise of mucormycosis cases was attributed to high doses of corticosteroids used to treat COVID-19 infection, which might have proven to be a double-edged sword [9].

Underlying immunocompromised states play a major role in developing invasive fungal diseases. Of these, diabetes mellitus is the most common immunocompromised state seen in patients with ROCM [10]. It was the same scenario in our study as well. Most of the patients admitted with ROCM were long-standing diabetics or recently diagnosed after COVID-19 sequelae. One other reason for the immunocompromised state among the patients coming to our centre was the prevalence of rampant use of steroids given elsewhere, in the management of COVID-19 in the early stages of the pandemic. 

Demographically, males are more commonly affected by ROCM than females, with an affected rate of 80.4% as compared to females in our study. This observation aligns with findings described in the literature [10].

Cross-sectional imaging in the form of CT and MRI plays a pivotal role in the diagnosis and preoperative mapping of ROCM. CT scans provide better bony delineation to pick up early erosion of adjacent facial bones, whereas MRI provides critical information regarding the soft tissue extension of the disease [11]. Both imaging modalities play a synergistic role and are essential in determining the spread and extent of the disease in the preoperative period. However, our data show that it may under-detect early disease in specific anatomical subregions.

Paranasal sinuses were the most common area of involvement with ROCM. Overall, maxillary sinus involvement (either unilateral or bilateral) was observed in 95 of 98 patients (96.9%), making it the most commonly involved sinus, followed by the ethmoid sinuses. Similar findings have also been obtained in recent studies [12]. Radiology demonstrated more than 90% sensitivity and 100% specificity for all spaces except for the left frontal sinus. This can be attributed to altered signals mimicking the collection or failure to detect subtle changes in the frontal sinus, which can be picked up with endoscopy. The chi-square analysis further demonstrated that the difference between radiological and endoscopic detection across the major sinuses was not statistically significant (p>0.5 for all sites), reinforcing that radiology and endoscopy generally correlate well in sinus disease mapping (as seen in Table 1). Nonetheless, the lower negative predictive values emphasise that a radiologically normal-looking area does not exclude early mucosal disease.

The middle turbinate was the most commonly involved among the three turbinates, as also shown in prior studies [13], whereas the superior turbinate was less commonly involved than the sinuses with ROCM in our study. The positive predictive value for superior turbinate involvement was 89.65% in the study, implying that even if radiologically we suspect the presence of the disease, we may not always find so on endoscopic exploration. The sensitivity of radiological findings to pick up the disease was low at 85.2%. This means that radiology might not be a useful tool to detect the involvement of the superior turbinate, and hence, routine exploration during surgical intervention of this area is warranted owing to the invasiveness of the disease. This finding was reinforced statistically - superior turbinate correlation between radiology and endoscopy, also showing no significant difference (p=1.000), suggesting that the observed discrepancies are not systematic but rather intrinsic to the anatomical subtleties and limitations of cross-sectional imaging in this region.

Soft-tissue delineation with MRI is warranted, particularly when orbital involvement is suspected. CT findings alone should not be relied upon for assessing orbital or early soft-tissue disease in ROCM, as subtle angioinvasive spread may be missed. Diffusion-weighted imaging further aids in differentiating infarction from abscess and can influence surgical decision-making [14]. Although MRI demonstrated good overall concordance with endoscopic findings on chi-square analysis, its sensitivity for early involvement of the superior extra-ocular muscle and intraconal compartments was comparatively lower, highlighting a limitation of radiological assessment in detecting subtle early orbital disease. Radiological findings should therefore be interpreted cautiously, and the absence of overt orbital signs or non-significant imaging comparisons should not be used to exclude early orbital involvement; targeted endoscopic evaluation or surgical exploration should be considered when suspicion persists.

Soft tissue involvement beyond the paranasal sinuses was less frequent than sinonasal disease but was still observed in a substantial proportion of cases. The spread beyond the sinuses happens along the neurovascular bundles without bony destruction [15]. MRI is the investigation of choice for other soft tissue involvement in ROCM. False positive cases in the study were seen with the pre-maxillary fat region, resulting in the positive predictive value as low as 28.6%. Subtle early edema during subcutaneous tissue involvement may give findings on sensitive diagnostic tools like MRI. However, as no false negative values were found, exploring the area of involvement picked up on MRI is always safer than leaving the disease to progress. 

MRI has proven useful for other critical soft tissue areas like the pterygopalatine fossa, pterygomaxillary fissure, retroantral pad of fat, and infratemporal fossa. Such areas are less likely to be explored during surgical intervention if not shown involvement in preoperative scans. The p-values for these maxillofacial soft-tissue spaces in our study were above the significance threshold, indicating that no statistically demonstrable difference was detected. However, this does not negate the practical concern of low radiological sensitivity observed in these regions. In our study, thus, a negative radiological scan cannot rule out the presence of disease in these areas and warrants endoscopic exploration of the site to look for hidden disease during surgery.

Overall, despite broad radiology-endoscopy concordance on chi-square analysis, reduced sensitivity in select anatomical subregions discussed earlier supports targeted operative exploration of these areas, even when preoperative imaging findings are non-contributory.

This retrospective study relied on previously recorded clinical data, which may introduce minor selection or documentation biases. As the study was conducted in a single tertiary-care centre, the findings may reflect local patient characteristics and practice patterns, and broader validation through multi-centre studies would be beneficial. Blinding of radiologists and surgeons was not feasible within the retrospective design; however, future prospective studies incorporating observer blinding and inter-observer reliability assessment could further strengthen diagnostic accuracy. As ROCM is a rapidly progressive disease, minor variations in the interval between imaging and surgical intervention were unavoidable, but these intervals were short and not expected to meaningfully affect the overall radiology-endoscopy correlation. Standardisation of imaging techniques and protocols across institutions may also enhance comparability and reproducibility in future research.

## Conclusions

ROCM is a rapidly progressive and potentially fatal invasive fungal disease associated with significant morbidity and mortality. Despite advances in medical therapy, timely diagnosis and surgical debridement remain central to management, alongside systemic antifungal treatment. Cross-sectional imaging with CT and MRI plays an important role in the preoperative assessment of ROCM by delineating disease extent, particularly in regions where spread may occur without overt bone erosion. MRI is especially valuable in evaluating suspected orbital involvement and soft tissue spread. However, imaging sensitivity is not uniform across all intra-orbital compartments, and early disease involvement in certain anatomical regions may be under-represented.

CT and MRI should therefore be regarded as complementary preoperative mapping tools rather than sole determinants of disease extent. Endoscopic correlation remains essential, particularly for assessing anatomically concealed areas and early mucosal involvement that may not be reliably detected on imaging. These findings underscore the importance of integrating radiological assessment with systematic endoscopic evaluation to optimise surgical decision-making in ROCM.
